# Chest Compression in Neonatal Cardiac Arrest: Cerebral Blood Flow Measurements in Experimental Models

**DOI:** 10.3390/healthcare8010017

**Published:** 2020-01-10

**Authors:** Anne Lee Solevåg, Po-Yin Cheung, Georg M. Schmölzer

**Affiliations:** 1 Department of Paediatric and Adolescent Medicine, Akershus University Hospital, 1478 Lørenskog, Norway; 2 Division of Paediatric and Adolescent Medicine, Oslo University Hospital, 0424 Oslo, Norway; 3 Neonatal Research Unit, Centre for the Studies of Asphyxia and Resuscitation, Royal Alexandra Hospital, Edmonton, AB T5H 3V9, Canada; poyin@ualberta.ca (P.-Y.C.); georg.schmoelzer@me.com (G.M.S.); 4 Department of Pediatrics, University of Alberta, Edmonton, AB T6G 1C9, Canada

**Keywords:** newborn infant, asphyxia, chest compression, animals

## Abstract

The main aim of this paper was to provide an overview of studies that measured cerebral blood flow (CBF), directly or indirectly, during chest compression (CC) in neonatal animals. Our main research question was: how did different ways of performing CC influence CBF. We also aimed to discuss strengths and limitations of different methods for measuring CBF. Based on a search in Medline Ovid, we identified three studies in piglets that investigated different CC:ventilation (C:V) ratios, as well as three piglet studies investigating continuous CC with asynchronous ventilation. CBF was measured indirectly in all studies by means of carotid artery (CA) flow and regional cerebral oxygenation (rcSO_2_). The CA provides flow to the brain, but also to extracerebral structures. The relative sizes of the internal and external carotid arteries and their flow distributions are species-dependent. rcSO_2_ is a non-invasive continuous measure, but does not only reflect CBF, but also cerebral blood volume and the metabolic rate of oxygen in the brain. Continuous CC with asynchronous ventilation at a CC rate of 120/min, and combining CC with a sustained inflation (four studies in piglets and one in lambs) provided a faster CBF recovery compared with the standard 3:1 C:V approach.

## 1. Introduction

Cerebral hypoxemia results in reduced cerebral vascular resistance and increased cerebral blood flow (CBF) [1]. This compensates for the decreased blood oxygen content during the initial phase of perinatal asphyxia. When the asphyxial process continues, cardiac output and systemic blood pressure falls and compensatory mechanisms fall short with resulting cerebral hypoxia-ischemia. Oxygen delivery to the brain depends on cerebral hemodynamics including CBF and cerebral blood flow velocity (CBFV). Blood oxygen content as determined by fraction of inspired oxygen (FiO_2_), pulmonary gas exchange and haemoglobin (Hb) concentration also influence cerebral oxygen delivery [2]. In cardiac arrest, assisted ventilation and chest compression (CC) is needed to maintain cerebral oxygen delivery [3].

The infrequent occurrence of neonatal cardiopulmonary resuscitation (CPR) with CC has impeded large randomized controlled clinical trials to identify approaches that optimize cerebral as well as systemic hemodynamics [4]. Thus, recommendations are based on mainly indirect evidence from adults, older children and animals, as well as expert opinion. Current guidelines recommend a 3:1 CC to ventilation (C:V) ratio with 120 events/min comprising 90 CC and 30 inflations [5].

As knowledge regarding the restoration of CBF following perinatal asphyxia is limited, there is an ongoing interest in studying CBF changes in perinatal asphyxia and resuscitation. Because of the lack of clinical data, the aim of this paper was to provide an overview of animal studies that studied CBF, directly or indirectly, during CPR with various approaches to CC. Our main research question was: how do different ways of performing CC influence CBF. We also aimed to discuss strengths and limitations of different methods for measuring CBF.

## 2. Materials and Methods

A search in Medline Ovid was performed July and December 2019 with the search words hypoxia AND cardiac massage AND infant, newborn AND animals. A search in Web of Science was performed December 2019 with a similar search strategy. Conference proceedings and the reference list of retrieved papers were hand searched for relevant researchers and papers. Publications were assessed based on title, abstract, and methods. Studies were included if they investigated neonatal animals that received CC. Studies were excluded when no CC was performed, or if they did not report measures of CBF.

## 3. Results

Due to the low number of studies in neonatal animals, we included some studies in non-neonatal animal models.

### 3.1. Cerebral Hemodynamic Measurements

Classic techniques used for measuring CBF under experimental conditions include xenon clearance [6], the Kety-Schmidt/nitrous oxide method [7], and radioactive- or fluorescent-labeled microspheres [8]. Newer methods include magnetic resonance imaging (MRI) and laser Doppler flowmetry. The latter is an invasive method for measurement of cortical microcirculation [9,10]. MRI and laser Doppler flowmetry can, similarly to microspheres, generally only take measurements from one animal at a time, or over short periods of time. This limits the number of animals that can be studied, as well the temporal resolution and measurement of dynamic CBF changes [11,12]. The most commonly used surrogate measures for CBF in animal models (Carotid artery (CA) flow and cerebral oximetry) are presented in the following, in addition to Doppler ultrasound that can also be used clinically.

#### 3.1.1. Doppler Ultrasound

CBFV obtained with Doppler ultrasound can be a good indicator of CBF, and CBF can theoretically be calculated from CBF and vessel cross sectional area. Transcranial Doppler ultrasound (TCD) is commonly used in clinical practice and provides continuous bedside measurement of CBFV in large arteries in a non-invasive manner [13]. However, TCD does not assess microvascular blood flow, and provides only an indirect measure of tissue perfusion [14]. TCD is also operator-dependent and 10%–30% of patients may lack a ultrasound transmission window [14,15,16].

The probe of the novel device NeoDoppler is placed over the anterior fontanel and provides real-time Doppler signals that include depth vs. time color M-mode, representing blood flow in several vessels at different depths, and the corresponding pulsed-wave Doppler curves from one specific depth. In a feasibility study (*n =* 25), NeoDoppler provided accurate and continuous CBFV data in several depths simultaneously in neonates at different gestational ages and with different disease conditions [17]. NeoDoppler measured a slightly lower pulsatility index than conventional pulsed-wave Doppler.

#### 3.1.2. Cerebral Oximetry

Near-infrared spectroscopy (NIRS) has been utilized in numerous CC studies of newborn animals [18,19,20]. NIRS is a non-invasive monitoring technique that measures regional cerebral oxygenation (rcSO_2_) continuously [21]. NIRS measures tissue Hb difference ([HbD]), defined as the difference between oxy-haemoglobin ([HbO_2_]) and deoxy-haemoglobin ([Hb]). [HbD] correlates with CBF [22]. However, [HbD] is also dependent on the balance between oxygen delivery and oxygen consumption, cerebral blood volume including arterial/venous volume ratio, and cerebral metabolic rate of oxygen [13].

Calculation of the concentration changes in oxygenated (ΔHbO_2_), deoxygenated (ΔHb), and total tissue haemoglobin (ΔcHb = ΔHbO_2_ + ΔHb) by the modified Beer-Lambert method has provided opportunity to utilize NIRS for cerebral perfusion measurement. Total Hb concentration change represents the change of cerebral blood volume within the tissue.

Diffuse correlation spectroscopy (DCS) is another optical technique that utilizes near-infrared light to measure microvascular CBF [23]. DCS is based on physical principles similar to those underlying NIRS. In contrast to NIRS, however, DCS provides a direct measure of CBF based on the statistical properties of the emerging light [23,24]. Combining traditional NIRS and DCS into one instrument creates a new opportunity to make comprehensive all-optical measurement of cerebral hemodynamics and cerebral oxygen metabolism. This so-called Hybrid DCS-NIRS technology was first introduced for cerebral monitoring in rats in 2001 [25] and in adult brain in 2004 [26,27].

#### 3.1.3. Carotid Artery Flow

Using ultrasonic measurement techniques, blood flow can be measured in real-time continuously. The common CA may distribute blood to the face and the brain at various extents. Attempts at determining the origin of intracerebral versus extracerebral blood flow have been made by occluding the external or internal CA circulations by e.g., ligation or thrombosis [28,29]. However, the relative sizes of the external vs. internal CA and communication distal to the experimental occlusion are species-dependent. In some species (e.g., sheep), blood flow from the common CA passes almost entirely to the external CA circulation and from there to both the face and brain [30]. In other species (e.g., man, pig), the internal CA dominates the afferent cerebral circulation [30,31]. The presence of a rete mirabile may further confound the association between common CA flow and CBF [28,29,31]. Although the extent to which the common CA flow reflects CBF may vary, Gratton et al. [32] demonstrated a direct correlation between CA flow and CBF during hypoxia and reoxygenation in lambs.

In piglets, about two-thirds of the flow to the brain go through the right common CA [28]. However, ligation of the right external CA of piglets does not quantitatively affect CBF [28] because of well-developed collateral circulation [9]. Nevertheless, temporary occlusion of the common CA leads to a substantial reduction in CBF, especially to the forebrain. In piglets, flow probe measurements in the unilateral common CA seemed to overestimate microsphere determinations of unilateral CBF by approximately 68% [28]. This overestimation likely reflects blood flowing through the right common CA to the right external CA to the scalp, face, and snout. The internal CA of piglets is deep and not easily accessible with unreliable signaling through ultrasonic measurement of the flow (personal experience). This may render the infrequent or rare reports of internal CA blood flow changes.

### 3.2. Cerebral Blood Flow During Different Ways of Performing CC

In asphyxiated piglets and lambs, different CC approaches have been investigated as alternatives to the standard 3:1 C:V ratio including different C:V ratios 2:1 [33], 4:1 [33], 9:3 [34], and 15:2 [35], continuous CC with asynchronous ventilations (CCaV) [36,37] at different CC rates (90/min, 100/min and 120/min) [38], and continuous CC with sustained inflation (SI) [39,40,41] at different CC rates [42] and SI duration [43].

#### 3.2.1. Different C:V Ratios

Animal studies suggest that different C:V ratios result in similar time to return of spontaneous circulation (ROSC), mortality, and systemic hemodynamic recovery [44].

Solevåg et al. demonstrated in asphyxiated piglets that a 9:3 (*n =* 16) [34] or 15:2 (*n =* 11) [35] C:V ratio did not result in improved median time to ROSC compared to a 3:1 C:V ratio (*n =* 16 and *n =* 11, respectively). There were no significant differences between the groups in the temporal changes in NIRS rcSO_2_, rcSO_2 actual_/rcSO_2 baseline_ or cerebral fractional tissue oxygen extraction. There was no significant difference in mean interleukin (IL)-1β in cerebrospinal fluid.

Pasquin et al. [33] randomized newborn piglets to four groups: 2:1 (*n =* 8), 3:1 (*n =* 8), or 4:1 (*n =* 8) C:V ratio; or sham (*n =* 7). The common CA flow of all intervention groups increased after hypoxia and both the 2:1 and 4:1 groups were significantly higher than the sham group. Common CA flow then decreased gradually during the reoxygenation period and was not different from that of the sham piglets by the end of the experiment. The rcSO_2_ of all experimental groups decreased significantly from baseline during hypoxia and then returned to the same level as the sham piglets’ immediately after resuscitation.

##### Non-Neonatal Animal Models

Traub et al. [45] resuscitated 2–3 weeks old piglets with potassium chloride-induced cardiac arrest and compared the C:V ratios 2:1, 3:1, 4:1, or 5:1. They reported no differences in the time to ROSC. Traub et al. [45] reported that the common CA had been cannulated in the piglets, but did not report direct or indirect CBF results.

The results of neonatal and non-neonatal animal studies suggest that the C:V ratio does not affect cerebral perfusion.

#### 3.2.2. Continuous Chest Compression with Asynchronous Ventilation

In two studies with a similar setup, with measurement of common CA flow and rcSO_2_, CCaV (CC rate 90/min) (*n =* 8 in both studies) resulted in similar time to ROSC compared to 3:1 C:V CPR (*n =* 8 in both studies) [36,37]. However, CCaV resulted in a lower mean arterial blood pressure 15 min after ROSC, and a higher myocardial lactate compared with 3:1 C:V CPR [36]. Common CA flow was not different during 3:1 C:V CPR vs. CCaV [36]. In [37], asphyxia resulted in significantly lower common CA flow compared to normoxic baseline and to sham piglets (*n =* 4). Common CA flow of the asphyxiated groups was significantly lower compared to sham piglets throughout the recovery period.

Patel et al. [38] examined whether different CC rates during CCaV would change time to ROSC and survival in asphyxiated piglets. Hemodynamic recovery of CCaV with a CC rate 120/min (CCaV + 120) (*n =* 8) was significantly improved compared with CCaV + 90 (*n =* 8) and CCaV + 100 (*n =* 8). rcSO_2_ was similar in all three intervention groups at 10 min after ROSC. rcSO_2_ returned to baseline in the CCaV + 120 group, while in the CCaV + 100 and CCaV + 90 groups rcSO_2_ remained significantly reduced compared with sham piglets and their own baselines. The same pattern was seen for common CA flow. Piglets in the CCaV + 90 and CCaV + 100 had higher brain IL-1β and IL-6. Cortical lactate levels were significantly higher in the CCaV + 90 and CCaV + 100 groups compared with the CCaV + 120 group, which corresponded with plasma lactate levels at the end of the 4-h recovery period [38].

##### Non-Neonatal Animal Models

In adult pigs with ventricular fibrillation, a 15:2 C:V ratio was as effective as continuous CC without assisted ventilations when CC was interrupted for only 4 s to deliver the two inflations [46,47,48,49,50]. When a more realistic 16-s interruption of CC was applied to the model, continuous CC without assisted ventilations resulted in significantly better neurologically normal survival than a 15:2 C:V ratio [51]. Ewy et al. [52] examined 24 h neurological outcome comparing a 30:2 C:V ratio with a 16-s interruption to deliver the two inflations, with continuous CC without assisted ventilations. No CBF measures were reported, but neurological intact survival was better in the continuous CC group.

CCaV has been examined in 2 week-old piglets with ventricular fibrillation [53]. CCaV (*n =* 8: CC rate 60/min, 95% oxygen (5% nitrogen), peak inspiratory pressure 60 mmHg (82 cmH_2_O)) compared with a 5:1 C:V ratio (*n =* 8: CC rate 100/min, 100% oxygen, peak inspiratory pressure 25–30 mmHg (34–41 cmH_2_O)) did not enhance microsphere-measured cerebral perfusion.

The results of neonatal and non-neonatal animal studies suggest that CCaV only improves cerebral perfusion when the CC rate is >100/min. Neurological outcome might depend on algorithm compliance when a CC pause is made to deliver assisted ventilations.

#### 3.2.3. Continuous Chest Compression Superimposed by a Sustained Inflation (CC + SI)

Studies in asphyxiated piglets showed that combining CC with SI (CC + SI) improved ROSC compared with 3:1 C:V CRP [39,40,42,54]. Piglets resuscitated with CC + SI (CC rate 120/min) (*n =* 8) had a faster recovery of common CA flow than 3:1 C:V CPR (*n =* 8) [39]. CC rates of 90/min vs. 120/min during CC + SI and CC + SI with either 20 s or 60 s SI resulted in similar time to ROSC, survival rates, and respiratory parameters [42,43]. However, mean arterial pressure, percent change in ejection fraction, cardiac output, and common CA flow during CC were higher in the CC + SI 90/min group compared to CC + SI 120/min [42]. The changes in right forehead rcSO_2_ of CC + SI 90/min and 3:1 C:V CPR followed a pattern similar to the changes in common CA flow [40]. In the same piglet model, CC with SI 20 s (*n =* 8) and CC with SI 60 s (*n =* 8) were compared to a 3:1 C:V ratio (*n =* 8), and there was no difference in cerebral cortical concentrations of IL-1ß, IL-6 and IL-8 among the three groups after CPR and reoxygenation. Common CA flow of all intervention groups was markedly reduced at the end of reoxygenation, resulting in lower rcSO_2_ as compared with baseline, but with no difference between the groups.

When CC at a rate of 90/min superimposed by 30 s SI (*n =* 8) was compared with CC at a rate of 120/min superimposed by SI (*n =* 8), common CA flow of both treatment groups was significantly lower than the sham group (*n =* 6) at the end of asphyxia and shortly before ROSC. Immediately following resuscitation, the common CA flow returned to the baseline value where it was maintained for the rest of the experiment. Changes in rcSO_2_ in both experimental groups followed a similar pattern to that of the common CA flow [42]. When different durations of the SI (20 s (*n =* 8) vs. 60 s (*n =* 8)) were compared, there was no difference in IL-1ß, IL-6 and IL-8 in the cerebral cortex among all three experimental groups after CPR and reoxygenation [43].

Combining CC with SI (*n =* 7) did not improve recovery in lambs compared to 3:1 C:V CPR (*n =* 6) [41]. The main difference between the piglets and lambs were that the pigs were post-transitional, whereas the lambs were examined during cardiopulmonary transition. The lambs were monitored with a left CA flow probe, but not rcSO_2_. The mean left CA flow was comparable between the groups throughout the study period. The authors concluded that based on the low mean left CA flows (6 ± 2 mL/kg/min) during CC compared to baseline in utero values (25 ± 4 mL/kg/min), cerebral oxygen delivery is likely to be very low during resuscitation. However, upon ROSC, the mean left CA flows increased significantly (29 ± 11 mL/kg/min) with better oxygen content (17 mL O_2_/dL), thus improving oxygen delivery to the brain. Therefore, establishing ROSC quicker limits the period of hypoperfusion and subsequent reperfusion injury and may improve neurologic outcomes.

The results from neonatal animal studies suggest that combining CC with a sustained inflation results in a faster recovery of common CA flow compared with 3:1 C:V CPR. CC + SI with a CC rate of 90/min provided a higher CA flow than CC + SI with a CC rate of 120/min. Changes in rcSO_2_ follow a similar pattern to that of the common CA flow during CC + SI.

## 4. Discussion

In this review of animal studies, we found that continuous CC with asynchronous ventilation at a CC rate of 120/min and combining a CC with a sustained inflation provided an improved recovery of CBF compared with a 3:1 C:V ratio. CC + SI with a CC rate of 90/min provided a higher CA flow than CC + SI with a CC rate of 120/min. The current recommendation is to provide 90 CC per minute and our findings might indicate that, in the experimental setting, a higher CC rate may improve CBF recovery. Optimizing CC improves cerebral and myocardial perfusion. Cerebral perfusion is crucial to brain cell survival during CPR, whereas myocardial perfusion is needed to obtain ROSC. CCaV is more exhausting [55] and might impair myocardial perfusion compared to 3:1 C:V CPR [56]. In addition, a CC rate of 120/min is more fatiguing than lower rates [57], which potentially affects CC quality in the clinical setting. With regards to sustained inflation, the randomized controlled Sustained Aeration of Infant Lungs (SAIL) trial was terminated due to more deaths within 48 h in the most immature infants (gestational age 23–24 weeks) that received a 15 s SI with a peak pressure of 20 cmH_2_O followed by a second 15 s SI with a peak pressure of 25 cmH_2_O. The deaths were not believed to be attributed to air leak, and only three infants had a confirmed intraventricular haemorrhage, although a cranial ultrasound examination was performed in only eight of 19 infants that died early. Thus, no specific cause of the increased early mortality was identified. The ongoing SURV1VE trial compares CC + SI (CC rate 90/min) with 3:1 C:V CPR in the delivery room, and is only recruiting infants >28 weeks’ gestation in order to reduce potential harm [58] (https://clinicaltrials.gov/ct2/show/NCT02858583).

In order to preserve cerebral viability, a minimum of 20% of normal CBF is needed [59,60]. CBF autoregulation is defined as maintenance of a constant intracranial blood flow despite changes in perfusion pressure [61]. In normotensive newborns, the perfusion pressure varies between 30 and 110 mmHg. Under and over these limits, autoregulation fails [62]. Cerebral autoregulation is influenced by pO_2_ and pCO_2_, with pCO_2_ being perhaps a more important determinant of CBF than mean arterial blood pressure [63]. NIRS has been used to study cerebral autoregulation in newborn infants [64]. There is a positive correlation between rcSO_2_ and pCO_2_ and a negative correlation between fractional tissue oxygen extraction and pCO_2_, and autoregulation may be compromised in severe asphyxia. If cerebral autoregulation is impaired in the asphyxiated infant, the risk of brain injury resulting from blood pressure changes caused by CC might outweigh the benefits of improved coronary and systemic perfusion. CCaV with a CC rate of 120/min and CC + SI improved hemodynamic recovery including CBF. Whether this is beneficial to the post-asphyxic neonatal brain remains undetermined. The focus of CC research has been on systemic and coronary hemodynamics with the primary endpoint often being ROSC. Little attention has been given to the effects of CC alone on cerebral circulation and neurological outcomes in asphyxiated infants.

In this review, CBF was measured indirectly in all studies by means of CA flow and rcSO_2_. Both methods have inherent sources of error, which might partly explain the lack of difference between the other CC interventions. The availability of studies is low, and non-neonatal animal data have limited applicability to birth asphyxia and delivery room resuscitation. It is stated in the 2015 American Heart Association Guidelines Update for Cardiopulmonary Resuscitation and Emergency Cardiovascular Care that: “A 3:1 compression-to-ventilation ratio is used for neonatal resuscitation where compromise of gas exchange is nearly always the primary cause of cardiovascular collapse” [5]. The non-neonatal animals included in this review had cardiac arrest induced by ventricular fibrillation, not asphyxia. Moreover, some of the older studies used exceedingly high airway pressures during CPR, and the generalizability of the results is limited even to older age groups. Thus, it may not be justified to extrapolate evidence from these studies to perinatal transition.

The first suggestion of cerebral hyperperfusion after birth asphyxia was obtained in 1979 when reduced pulsatility in the anterior cerebral artery was demonstrated with Doppler ultrasound. After a phase of hyperperfusion (cerebral hyperaemia), delayed cerebral hypoperfusion develops partly due to increased cerebrovascular resistance. Measuring cerebral perfusion during CPR and after ROSC is clinically relevant. Feasible measurement techniques have not been readily available, but are emerging. Research should focus on the relationship between cerebral hypo- and hyperperfusion and neurological outcome, and developing treatment guidelines.

## 5. Conclusions

In conclusion, despite the fact that systemic hemodynamic recovery was different between different CC methods, only two studies found a difference in common carotid artery flow in favor of continuous chest compression with asynchronous ventilation, and continuous chest compression superimposed by a sustained inflation. Improved methods for measuring cerebral blood flow in the experimental and clinical setting is needed and under way. The association between cerebral blood flow during and after cardiopulmonary resuscitation, and short- and long-term neurological outcome should be examined.

## Abbreviations

CBF	cerebral blood flow;
CBFV	cerebral blood flow velocity;
FiO_2_	fraction of inspired oxygen;
Hb	haemoglobin;
CC	chest compression;
CPR	cardiopulmonary resuscitation;
C:V	Compression:Ventilation;
CA	carotid artery;
TCD	Transcranial Doppler ultrasound;
NIRS	Near-infrared spectroscopy;
rcSO_2_	regional cerebral oxygenation;
[HbD]	haemoglobin difference;
[HbO_2_]	oxy-haemoglobin;
[Hb]	deoxy-haemoglobin;
DCS	Diffuse correlation spectroscopy;
ROSC	return of spontaneous circulation;
IL	Interleukin;
CCaV	continuous chest compression with asynchronous ventilations;
SI	sustained inflation
